# Identifying vascular stiffening-sensitive macrophages through integration of single-cell transcriptomics and imaging flow cytometry

**DOI:** 10.52601/bpr.2025.240063

**Published:** 2025-12-31

**Authors:** Jin Wang, Jiyin Wang, Yuting Zhang, Chaoyang Xiong, Shun-Ai Liu, Yaxian Kong, Jing Zhou, Xi Wang

**Affiliations:** 1 National Key Laboratory of Intelligent Tracking and Forecasting for Infectious Diseases, Beijing Ditan Hospital, Capital Medical University, Beijing 100015, China; 2 Beijing Key Laboratory of Emerging Infectious Diseases, Institute of Infectious Diseases, Beijing Ditan Hospital, Capital Medical University, Beijing 100015, China; 3 Beijing Institute of Infectious Diseases, Beijing 100015, China; 4 National Center for Infectious Diseases, Beijing Ditan Hospital, Capital Medical University, Beijing 100015, China; 5 Department of Physiology and Pathophysiology, School of Basic Medical Sciences, State Key Laboratory of Vascular Homeostasis and Remodeling, Department of Cardiology and Institute of Vascular Medicine, Peking University Third Hospital, National Health Commission Key Laboratory of Cardiovascular Molecular Biology and Regulatory Peptides, Beijing Key Laboratory of Cardiovascular Receptors Research, Peking University, Beijing 100191, China

**Keywords:** Vascular stiffening, Single-cell transcriptomics, Imaging flow cytometry, SPP1, Macrophage

## Abstract

Increased extracellular matrix (ECM) stiffness, a hallmark of risk in cardiovascular disease (CVD), is closely associated with inflammation triggered by immune cell infiltration in the vessel wall. While numerous immunotherapies targeting inflammation in CVD are being developed, the specific immune components and key factors that respond to arterial stiffness remain unclear. In this work, we analyzed single-cell transcriptomics to identify immune cell populations sensitive to mechanical stress in stiffened carotid plaques. We utilized an *in vitro* model of polyacrylamide gels with varying stiffness and an *in vivo* mouse model of acute calcification to replicate arterial stiffening. An imaging flow cytometry panel was employed to determine specific cell populations and gene expression in response to ECM stiffening. The scRNA-seq analysis revealed that SPP1^high^ macrophages constitute a prominent myeloid population influencing extracellular matrix composition. We uncovered that macrophages exhibit elevated SPP1 protein levels when cultured on a stiffer matrix. Additionally, the percentage of SPP1^high^ macrophages increased in the stiffened arterial wall in the mouse model of vascular calcification. Collectively, we combined single-cell transcriptomics analysis with *in vitro* imaging flow cytometry studies to identify SPP1^high^ macrophages as a population sensitive to ECM stiffness. Our findings suggest that macrophage SPP1 could serve as a potential biomarker for patients experiencing arterial stiffening.

## INTRODUCTION

Vascular stiffness is a mechanical hallmark in the progression of various cardiovascular diseases including coronary artery disease, myocardial infarction, stroke and peripheral artery disease (Chirinos *et al.* 2019). The increased wall stiffness has been attributed to extracellular matrix (ECM) remodeling including ECM protein deposition/degradation and cross-linking (Lacolley *et al.* 2020). Under physiological conditions, the stiffness of the arterial wall is approximately 2–5 kPa, while in the diseased area, atherosclerotic plaques for example, can reach up to 96 kPa (Rezvani-Sharif *et al.* 2019). Vascular stiffening is closely associated with the local inflammation triggered by immune cell infiltration in the vessel wall, including macrophages, T cells and NK cells (Engelen *et al.* 2022). We have previously reported that the cross-talk between macrophages and vessel wall cells orchestrates vascular inflammation, with the pro-inflammatory microenvironment further exacerbating arterial stiffness (Wang *et al.* 2022). This suggests a macrophage-mediated mechanism through which inflammation influences arterial stiffness. With the development of single-cell RNA sequencing, the diversity and heterogeneity of the phenotype of infiltrated macrophages in the vessel wall have been identified (Chen *et al.* 2024). However, the key subtype of macrophages that senses and responds to vascular stiffening remains unclear.

Secreted phosphoprotein 1 (SPP1), also known as osteopontin, was initially discovered in cells involved in bone morphogenesis. With its potential role in driving collagen deposition and organization (Ding *et al.* 2024; Li *et al.* 2024), SPP1 regulates various pathological events associated with extracellular matrix (ECM) remodeling, including calcification (Grau *et al.* 2012), tumor progression (Matsubara *et al.* 2023) and fibrosis (Hoeft *et al.* 2023; Song *et al.* 2021). SPP1 has been reported as a potential biomarker for vascular disease, as abnormal deposition and modification of SPP1 can be detected in the calcified vessel walls. Furthermore, serum SPP1 levels are increased in patients with coronary artery disease (Huang *et al.* 2024). Fu *et al.* revealed that SPP1^+^ macrophages accumulate in perivascular adipose tissue (PVAT) and exacerbate fibrosis by interacting with fibro adipogenic progenitor cells, thereby confirming the correlation between SPP1^+^ macrophages and fibrosis in CVD. However, the molecular mechanisms regulating SPP1 expression in macrophages remain unknown.

In this study, we analyzed single-cell transcriptomics from specimens obtained from patients undergoing carotid endarterectomy. We developed an *in vitro* model using polyacrylamide gels with varying stiffness, and an *in vivo* mouse model of acute calcification to simulate arterial stiffening. A method integrating bioinformatics analysis and imaging studies was established to identify mechanosensitive populations of plaque infiltrated macrophages. Our findings indicate that SPP1^high^ macrophages are sensitive to ECM stiffness, and that the expression and secretion of SPP1 are influenced by substrate stiffness. Our study elucidates the mechanism by which SPP1 levels increase with the progression of vascular stiffening.

## RESULTS

### Immune cell atlas in human carotid plaques

**Figure 1:**
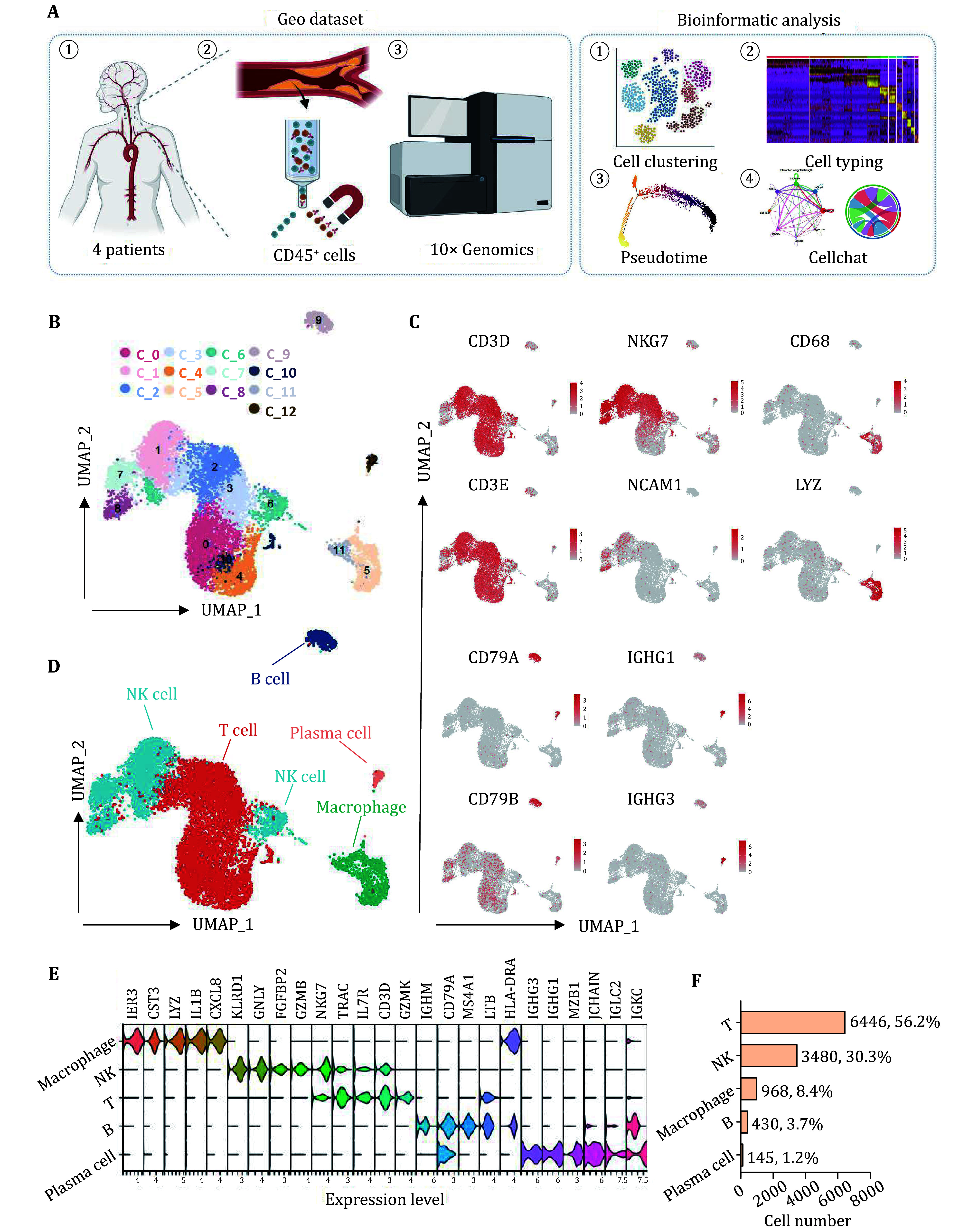
Immune cell distribution in human atherosclerotic plaques. **A** Workflow of the study including the raw data acquisition and bioinformatic analysis. **B** The Uniform Manifold Approximation and Projection (UMAP) plots of all cells which were divided into 13 clusters namely from C_0 to C_12. **C** The featured plot of classical marker genes of T cells (CD3D, CD3E), NK cells (NKG7, NCAM1), macrophages (CD68, LYZ), B cells (CD79A, CD79B) and plasma cells (IGHG1, IGHG3). **D** Profiles of the UMAP plots with each color represented one cell type. **E** Expression of top 5 marker genes of each cell type. **F** An overview of the number and percentage of each cell type

To determine the cellular composition of human diseased plaque areas, we analyzed single-cell RNA sequencing data from the GEO dataset (GSE235437). Briefly, carotid plaques from four patients undergoing CEA were digested to obtain single-cell suspension using a digestion protocol. CD45^+^ immune cells were selected to perform scRNA sequencing by using the 10× Genomics Chromium platform as previously described (Eberhardt *et al.* 2023). Bioinformatic analysis was performed to identify the immune cell atlas within the diseased vessel wall (Fig. 1A). 11,469 cells were obtained after quality control, followed by cell clustering. A total of 13 clusters were finally identified (Fig. 1B and supplementary Fig. S1A). We combined SingleR with manual annotation based on the expression of known markers to map the clusters to various cell types, designating them as T and NK cells, B cells, plasma cells, and macrophages (Figs. 1C and 1D, supplementary Fig. S1B). The top five markers in each cell type were identified using the “FindAllMarkers” function in Seurat (Fig. 1E and supplementary Fig. S1C). The T cell cluster constituted the largest population, followed by the NK cell, Macrophage, B cell and plasma cell (Fig. 1F).

### Intercellular communication and molecular interaction networks in the carotid plaque

**Figure 2:**
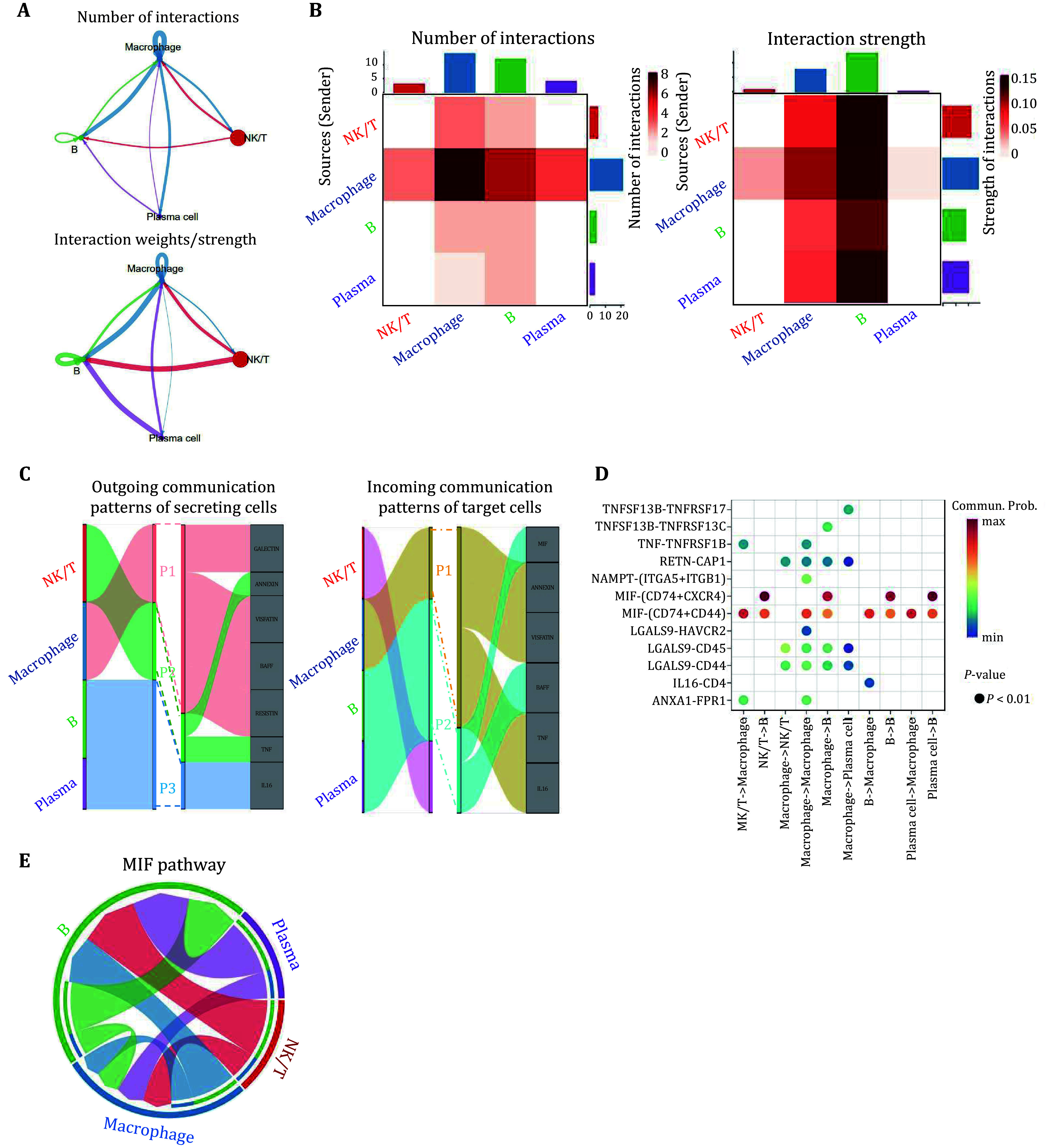
Analysis of cell-cell communication using CellChat. **A** Chord diagrams showing the cell-cell interaction between different cell types. The upper diagram showing the number of interactions whereas the lower showing the interaction strength. **B** Heatmap showing the number of interactions (left) and interaction strength (right) between all cell types in carotid plaques. **C** Alluvial plots showing cellular communication patterns in the carotid plaques including outgoing communication patterns (left) and incoming communication patterns (right). The thickness of the flow indicates the contribution of the cell type or signaling pathway to each pattern. **D** Dot plot showing the ligand-receptor pairs with significant probabilities among all cell types. **E** Chord diagram of MIF signaling networks in the carotid plaques

To investigate the cell–cell interaction network among the cell types identified in our work, the Cellchat analysis was performed. Macrophages exhibited high interaction counts and strength with other cell types (Figs. 2A and 2B). The CellChat method also uses a pattern recognition approach based on non-negative matrix decomposition to identify global communication patterns as well as key signals in different cell types (Zhao *et al.* 2022). There are three patterns for outgoing signals and two for incoming signals (Fig. 2C). The outgoing signaling from macrophages was characterized by pattern 1 (P1), which includes GALECTIN, VISFATIN, BAFF, RESISTIN pathways, while T/NK cells were characterized by pattern 2 (P2) including ANNEXIN and TNF pathway. The outgoing signaling of B and NK cells was defined by IL16, which belongs to pattern 3 (P3). The incoming communication pattern for macrophages was pattern 1, containing the ANNEXIN, VISFATIN, TNF and IL16 pathways. The incoming signaling of B/Plasma cells was characterized by pattern 2 (P2), which includes MIF and BAFF. We subsequently performed receptor-ligand pair analysis among all cell types and noted that MIF signaling, essential for macrophage function (Calandra and Roger 2003), was the predominant signaling pathway in cell-cell interaction (Figs. 2D and 2E). These results suggest that macrophages play a central role in cellular cross-talk within the carotid plaque.

### Heterogeneity of carotid plaque infiltrated macrophages

**Figure 3:**
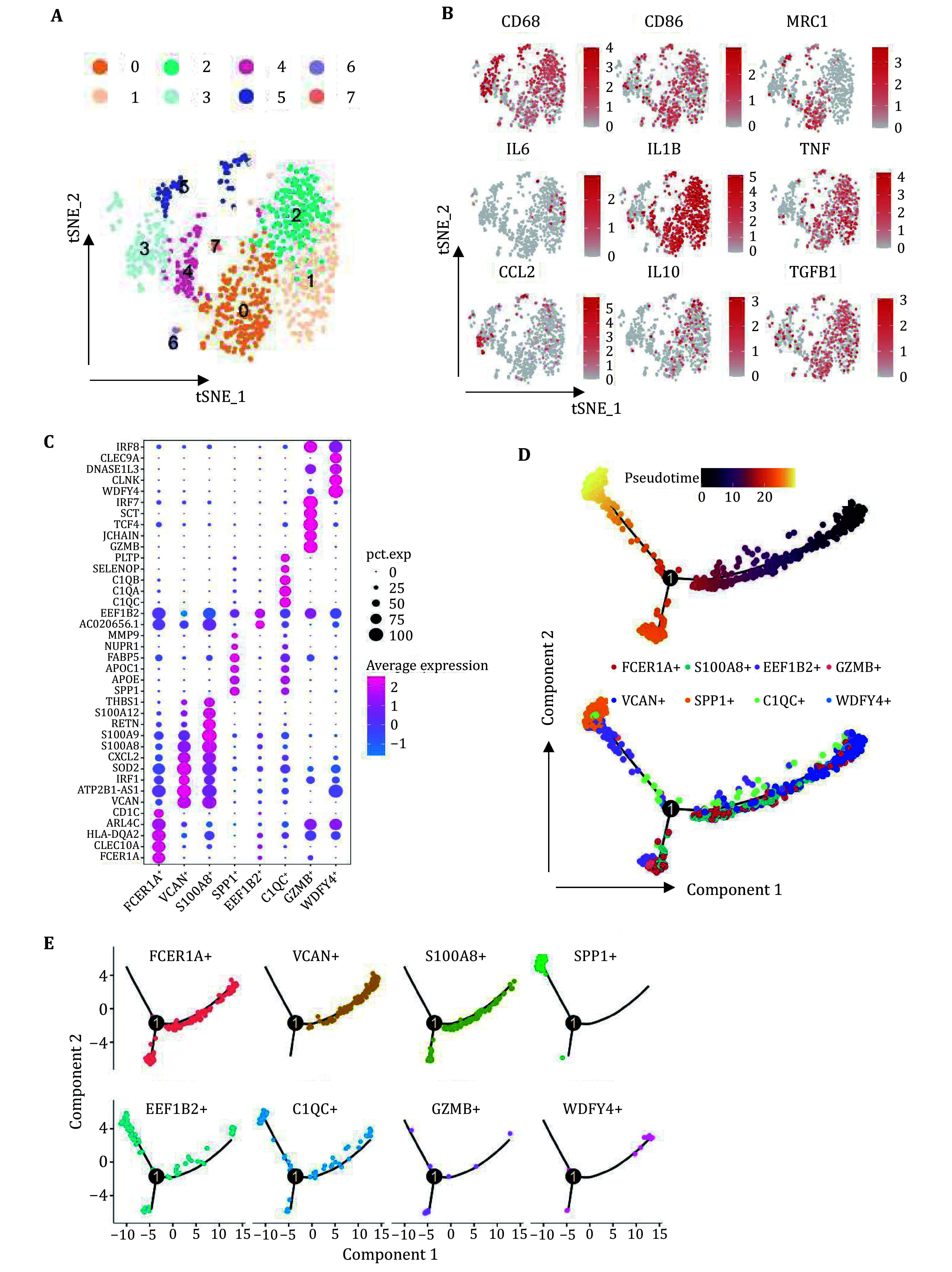
Identification of macrophage subpopulation and differentiation trajectory. **A** T-distributed neighbor embedding (t-SNE) plot of individual macrophages. The color represents the macrophage subtype. **B** Feature plots showing classical marker genes of macrophages (CD68, CD86, MRC1), proinflammatory genes (IL6, IL1B, TNF, CCL2) and anti-inflammatory genes (IL10, TGFB1). **C** Dot plot showing the top marker gene expression in each subcluster. **D**,**E** The dynamics of the macrophages subtype as shown by the monocle 2 trajectory plot

We further identified subtypes of macrophages. The cells were subclustered into eight populations (Fig. 3A), the expression of classical macrophage markers, including the pan marker CD68, M1 marker CD86, M2 marker MRC1, proinflammatory genes IL6, IL1B, TNF, CCL2 and anti-inflammatory genes IL10, TGFB1, was elucidated using feature plots (Fig. 3B). The dot plots displayed the expression of the top markers by each subtype (Fig. 3C). We labeled the subpopulations based on the highest expression of the leading gene in each cluster. Cell trajectory analysis was performed to elucidate the differentiation relationship among these eight subclusters, VCAN^high^ cells were located at the early stage of differentiation trajectory, while SPP1^high^ cells were situated at the terminal of differentiation trajectory. This evidence suggests that SPP1^high^ macrophages may perform specific biological functionsincarotid plaques (Figs. 3D and 3E).

**Figure 4:**
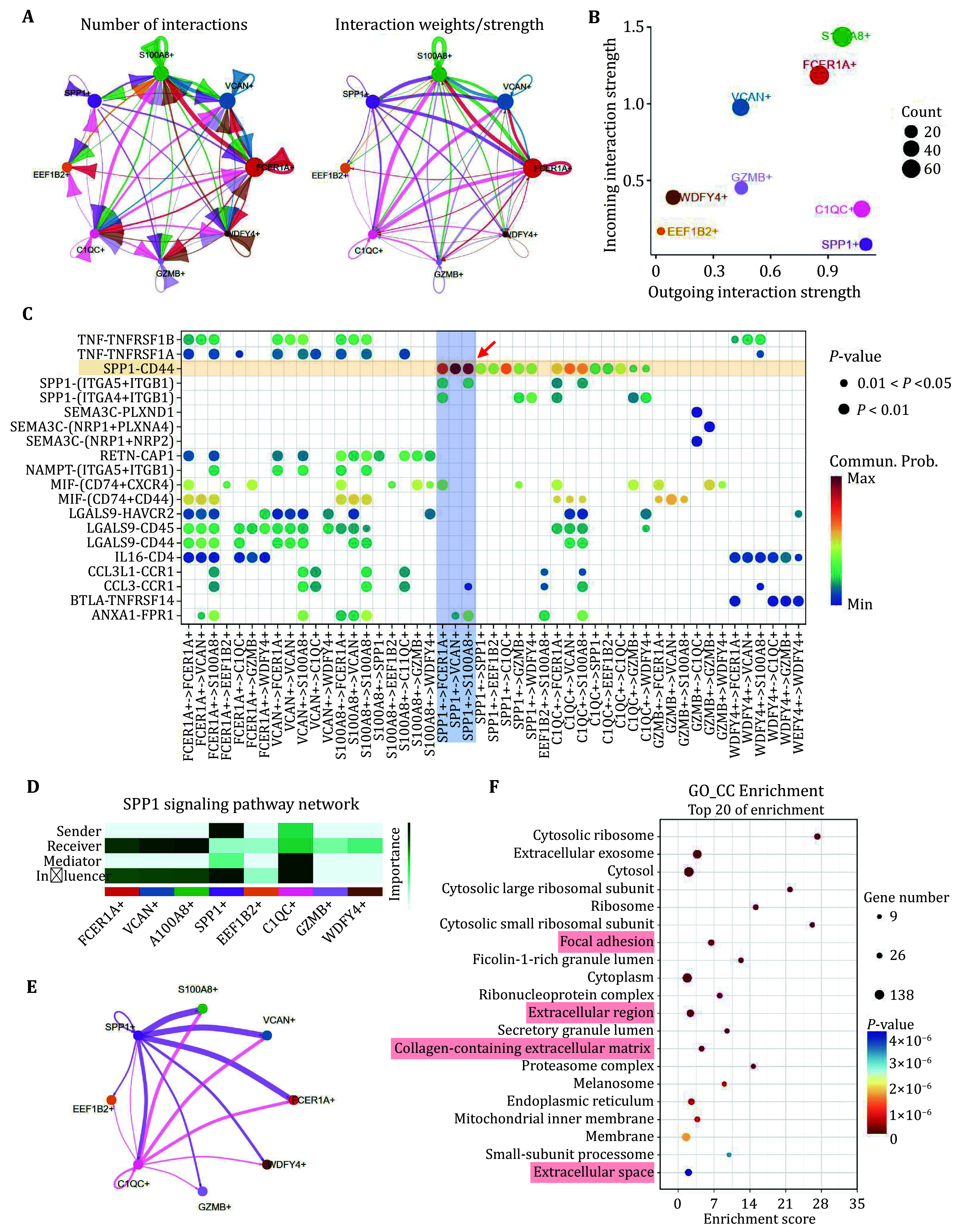
Macrophage secreted SPP1 mediates cell-cell communication in the plaque. **A** Interaction network constructed by CellChat. Thicker lines indicate more (left) and stronger (right) interaction with other types of cells. **B** The scatter plot shows the relative outgoing and incoming interaction strength of secreted signaling for each subtype of macrophages. **C** Ligand-receptor pairs are shown in the bubble plot. The red arrow indicates SPP1-CD44 signaling. **D** The heatmap showing the sender and receiver of SPP1 signaling in macrophages. **E** SPP1 signaling network constructed by CellChat. Thicker lines indicate stronger interaction between subtypes. **F** GO_CC enrichment analysis of DEGs in SPP1^high^ subtype. Highlight terms were ECM-related pathways

Cell–cell communication analysis was carried out to investigate receptor–ligand interactions among macrophage subclusters. The number and strength of interactions demonstrated versatile links between eight clusters (Fig. 4A), the SPP1^high^ subtype, exhibiting the highest outgoing interaction strength, primarily interacted with others through an “output” mode (Fig. 4B). We performed ligand-receptor pair analysis and identified the SPP1-CD44 interaction as the predominant pathway mediating communication between SPP1^high^ and other macrophage clusters (Fig. 4C). Furthermore, FCER1A^high^ (FCER1A^+^), VCAN^high^ (VCAN^+^) and S100A8^high^ (S100A8^+^) may serve as the receiver for SPP1 signaling (Figs. 4D and 4E). To explore the functions of plaque infiltrated SPP1^high^ macrophages, we analyzed differentially expressed genes (DEGs) in the SPP1^high^ cluster compared to other subclusters. A total of 108 genes were significantly upregulated in the SPP1^high^ cluster with a threshold of ︱log2Fc︱> 0.2. The top enriched KEGG pathway identified was “fluid shear stress and atherosclerosis”, confirming that SPP1^high^ macrophages are essential for vascular homeostasis (supplementary Fig. S2). We then performed GO_CC enrichment analysis to determine the subcellular compartments of DEGs (Fig. 4F). The extracellular-related GO terms in the cellular component categories, including collagen-containing extracellular matrix (ECM), were significantly enriched, indicating that SPP1^high^ macrophages may be involved in regulating ECM composition and structure. Our findings are consistent with the earlier reports that SPP1 contributes to ECM remodeling (Zhou *et al.* 2022).

### SPP1^+^ macrophages were enriched in the calcified aorta of mice

**Figure 5:**
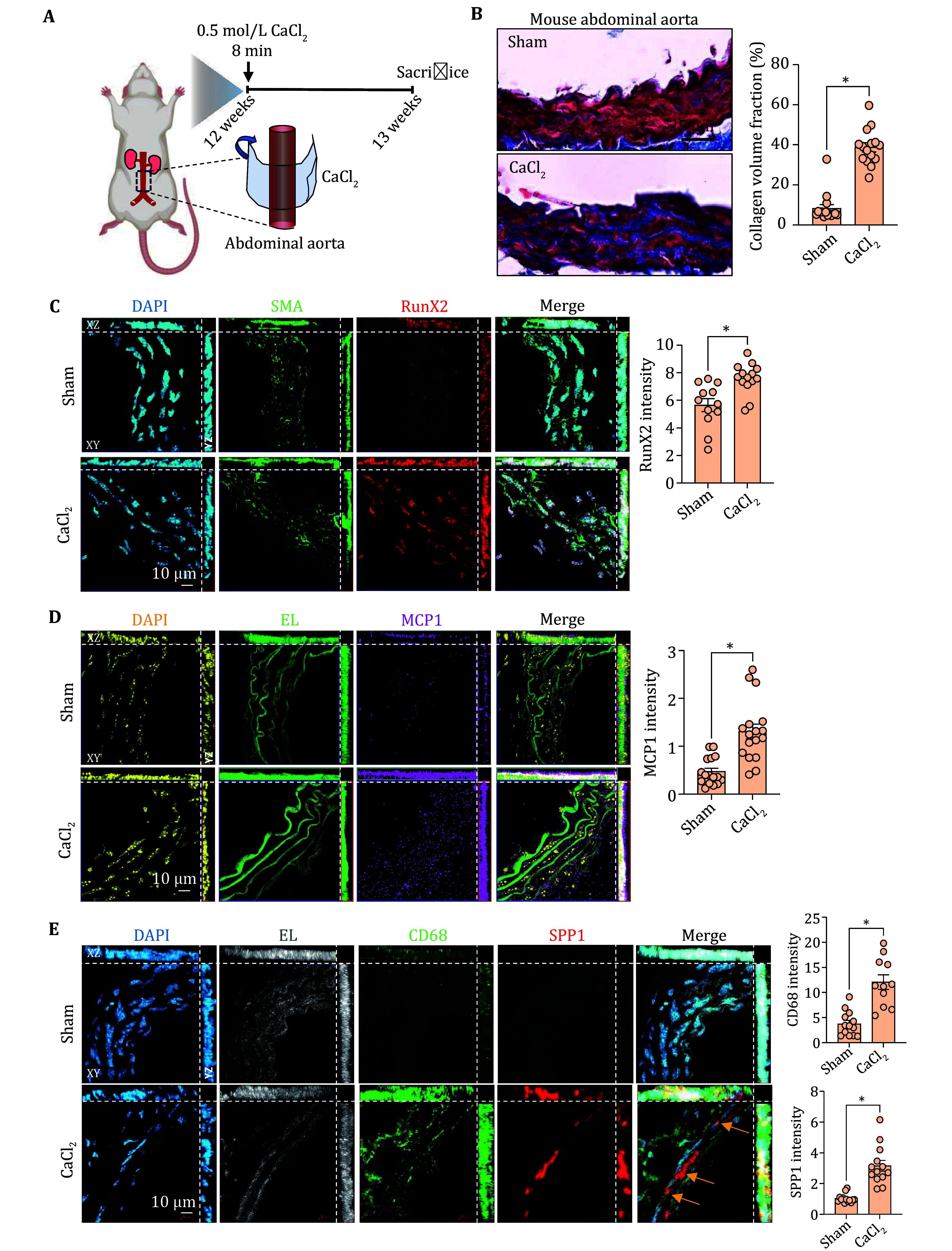
SPP1^high^ macrophages were increased in the diseased aorta. **A** Schematic diagram of the acute vascular injury model in mouse. **B** Representative Masson staining and the quantification of collagen volume fraction in the abdominal aorta from the CaCl_2_-incubated or sham-operated mice. **C** Representative immunofluorescence staining of SMA, RunX2 and quantification of RunX2 in mouse abdominal aorta with or without CaCl_2_-incubation. **D** Representative immunofluorescence staining and quantification of MCP1 in mouse abdominal aorta with or without CaCl_2_-incubation. **E** Representative immunofluorescence staining and quantification of CD68, SPP1 in mouse aortas with or without CaCl_2_-incubation. EL: Elastic Lamina. *P < 0.05 vs. the indicated group. One dot represents a value calculated from one field

To investigate the involvement of SPP1^high^ macrophages in the progression of vascular stiffening *in vivo*, we developed a CaCl_2_-induced arterial calcification/stiffening mouse model as previously described (Wang *et al.* 2022). Briefly, 12-week-old mice underwent local incubation of their abdominal aortas with 0.5 mol/L CaCl_2_ or saline for 8 min. The aortas were then harvested one week-post surgery for further assays (Fig. 5A). The vascular stiffening resulting from CaCl_2_ incubation was confirmed by the deposition of collagen (Fig. 5B). and increased expression of Runx2, an osteogenic differentiation marker of smooth muscle cell in the vascular wall (Fig. 5C). Furthermore, the intensity of MCP1, an inflammatory cytokine, was significantly increased in tunica media of the vessels subjected to CaCl_2_ incubation (Fig. 5D). Subsequently, we labeled the abdominal aortas with CD68 antibody, a pan marker for macrophages. The CD68 intensity increased following CaCl_2_ incubation, indicating enhanced macrophage infiltration, which is consistent with our previous findings (Wang *et al.* 2022). Additionally, the intensity of SPP1 was elevated in the CaCl_2_ incubated aortas (Fig. 5E). This finding corroborates with our bioinformatic predictions that SPP1^high^ macrophages may be sensitive to arterial stiffness.

### Imaging flow cytometry to identify SPP1 expression and secretion in macrophages

**Figure 6:**
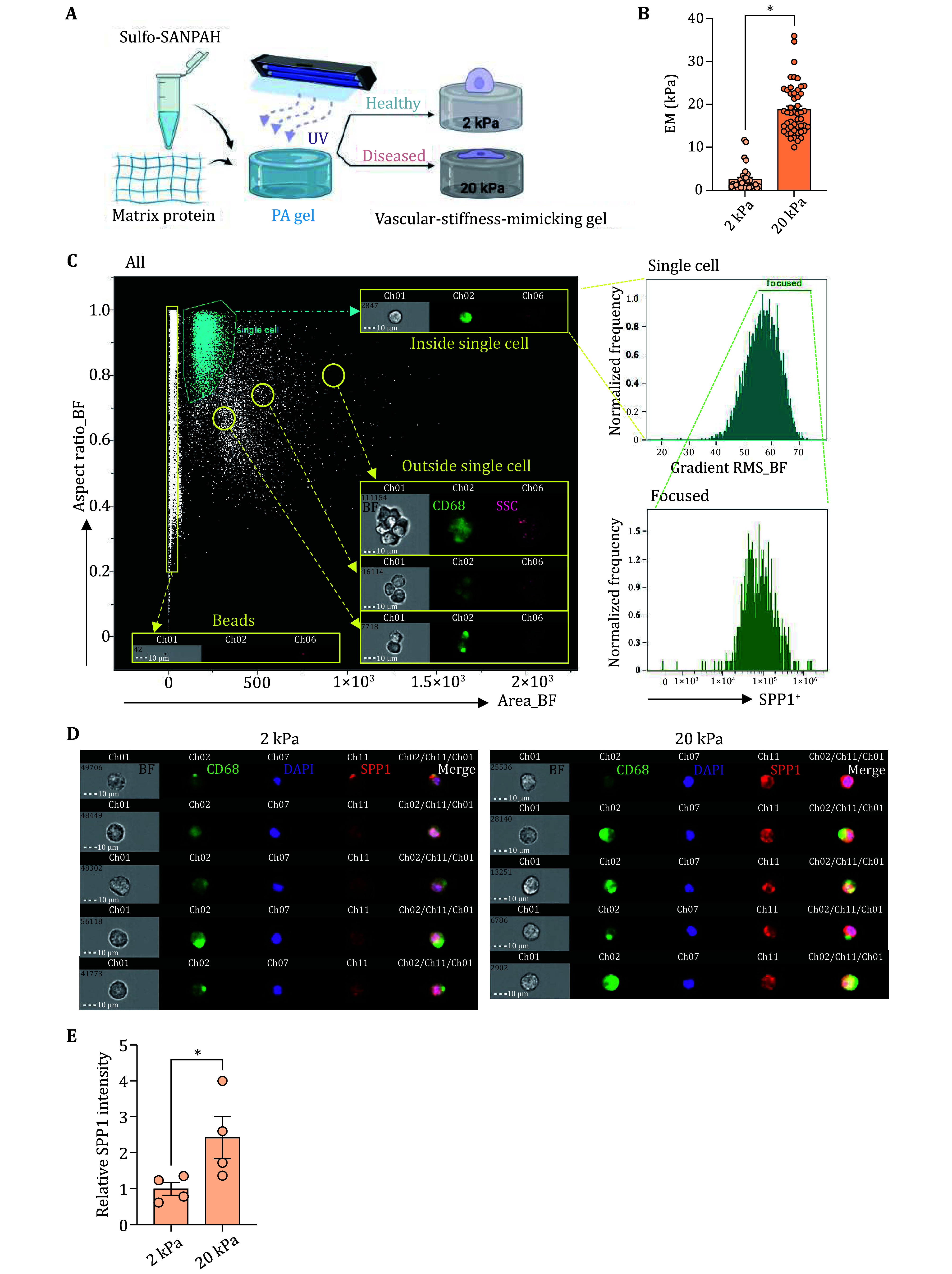
Imaging flow cytometry to identify SPP1 expression in macrophages. **A** Schematic diagram illustrating the *in vitro* model of substrate stiffening. PA gels were prepared with the combination of 40% acrylamide and 2% bis-acrylamide (Created with BioRender.com). **B** Elastic moduli of gels were measured by nanoindentation. **C** The area of the cell versus the aspect ratio of the minor axis divided by the major axis in the brightfield was used to exclude doublets and debris. The best focus was gated from the single cell gate. The fluorescence intensity of the SPP1 channel was measured under the focused gate. **D** Representative images of single, focused, SPP1^+^cells collected from 2 kPa vs. 20 kPa substrate. **E** Relative SPP1 fluorescence intensity of cells cultured on 2 kPa vs. 20 kPa substrate. *P < 0.05. Each dot represents an independent experiment

To investigate whether matrix stiffness modulates the population of SPP1^+^ macrophages *in vitro*, we cultured the Raw 264.7 mouse macrophage cell line on PA gels with different stiffness (Fig. 6A). 2 kPa and 20 kPa were selected to mimic healthy and diseased vascular stiffness, respectively, based on the physiological and pathological conditions of the vessel wall (Boutouyrie *et al.* 2021; Chirinos *et al.* 2019; Xie *et al.* 2018) (Fig. 6B). Additionally, we induced bone marrow-derived monocytes to macrophages (BMDM), the primary source of vascular-infiltrating macrophages (Chen *et al.* 2024), to mimic the *in vivo* diseased microenvironment. The cell area significantly increased when cultured on the stiffer matrix (supplementary Fig. S3A), consistent with previous reports (Xu *et al.* 2020), indicating the successful establishment of the *in vitro* matrix stiffening model. An imaging flow cytometry panel was set up to study the SPP1 expression in macrophages. Briefly, the area and aspect ratio (the ratio of the minor axis divided by the major axis) of the bright field cell images are used to gate single cells, whereas doublet and debris were excluded. Cells with the best focus were further gated according to the gradient RMS, which measures the sharpness quality of an image. The SPP1 protein level was determined from the fluorescence intensity of SPP1 channels of the best-focused cells (Fig. 6C). The SPP1 level was significantly higher in cells cultured on 20 kPa PA gel compared to the 2 kPa gel (Figs. 6D and 6E). The increased expression of SPP1 protein in the stiffer matrix was further validated through western blot assay and immunofluorescence in BMDM (supplementary Figs. S3B and S3C). Collectively, these data demonstrate that matrix stiffness promotes SPP1 protein expression in macrophages.

**Figure 7:**
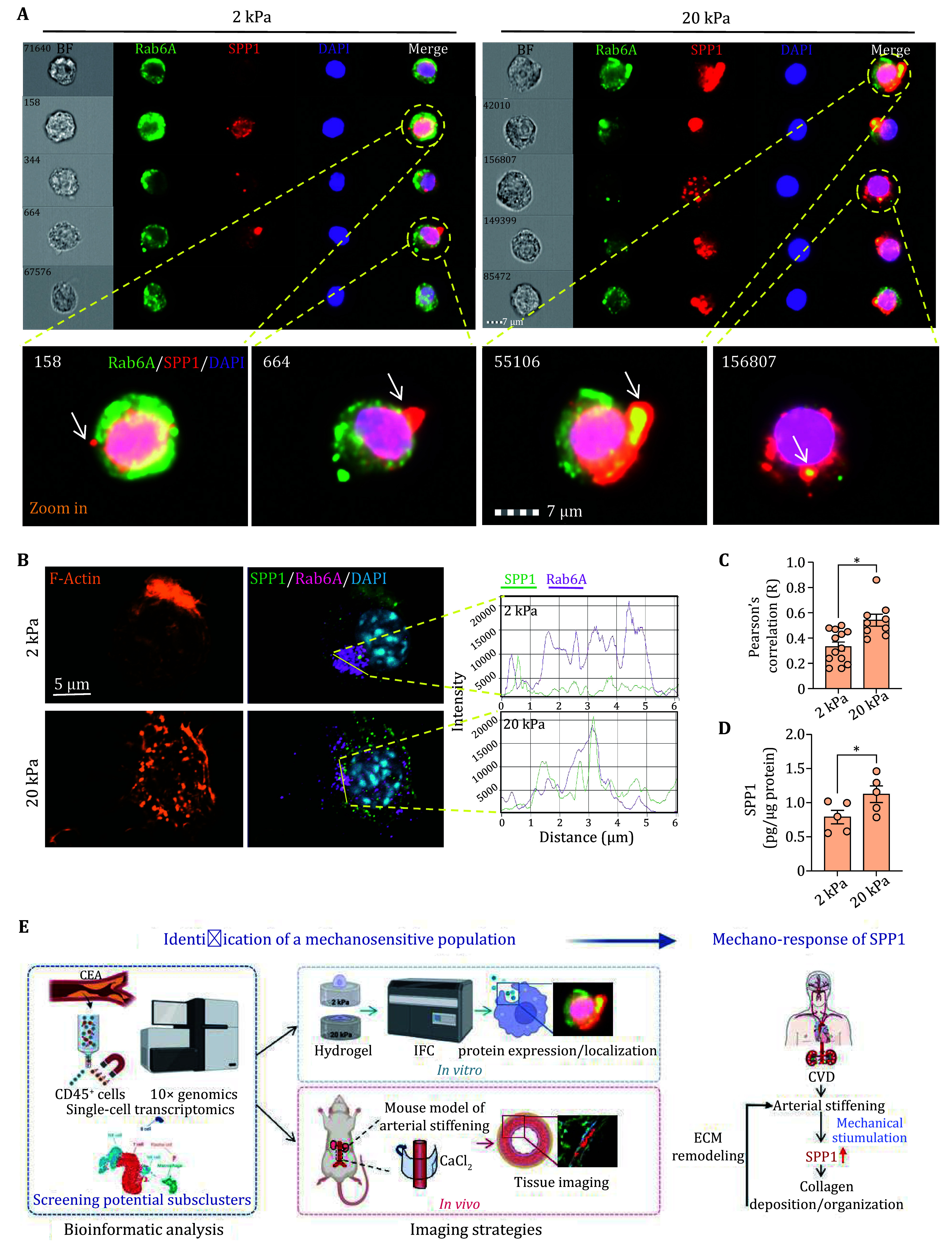
Substrate stiffening promotes SPP1 secretion. **A** Representative cells from imaging flow cytometry stained with DAPI, Rab6A and SPP1. The arrows on the zoom-in images show the SPP1 spots with an enhanced colocalization with Rab6A in the 20kPa group. **B** Left: representative images from a super-resolution microscope. Cells were stained with DAPI, Rab6A, SPP1 and F-actin. Right: the line plots show SPP1 and Rab6A co-localization of areas indicated by yellow arrows. **C** The colocalization of SPP1-Rab6A was analyzed by Pearson’s correlation coefficients. *P < 0.05. Each dot represents a field. **D** ELISA assay to determine SPP1 concentration in the culture media of RAW264.7 cells. *P < 0.05. Each dot represents an independent experiment. **E** Schematic graph of this work

To determine whether substrate stiffness regulates SPP1 protein secretion, we performed imaging flow cytometry to examine the co-localization of SPP1 and Rab6A, a RAB GTPase that modulates the constitutive secretory pathway and directly binds to SPP1 (Patwardhan *et al.* 2017; Yang *et al.* 2023). The SPP1^+^Rab6A^+^ double positive cells were gated, and we found that the co-localization of these two proteins was enhanced in macrophages cultured on stiffer substrates (Fig. 7A), which was confirmed by the super-resolution imaging (Figs. 7B and 7C). The concentration of SPP1 in the culture media of macrophages significantly increased from 0.79 to 1.12 pg/μg protein when cells were cultured on stiffer substrate (Fig. 7D), indicating elevated SPP1 secretion. Taken together, we identified that SPP1 expression and secretion are sensitive to substrate stiffness.

## DISCUSSION

The development of scRNA-seq has facilitated the identification of novel cell types and the characterization of gene expression profiles in specific cell populations (Paik *et al.* 2020; Tong *et al.* 2023). Our previous work indicated that arterial stiffening exacerbates the proinflammatory responses of vascular wall cells, as evidenced by cytokine production in vascular smooth muscle cells and immune cell infiltration within the arterial wall (Wang *et al.* 2022). In this study, we aimed to elucidate the mechano-response of immune cells to arterial stiffening by analyzing the scRNA transcriptomics of immune cell populations infiltrated into the stiffened vessel wall. We identified five distinct cell types, with T cells constituting the largest populations among all immune cells, confirming the inflammatory microenvironment of carotid plaques as previously described (Winkels *et al.* 2018). Furthermore, we found that genes expressed in SPP1^high^ macrophages were enriched in categories associated with collagen-containing cellular components, suggesting that SPP1^high^ macrophages may play a crucial role in regulating collagen homeostasis. Given that collagen is one of the major components of the ECM and provides structural and mechanical properties to tissue (Poulis *et al.* 2022), the abnormal ECM remodeling is regarded as a primary contributor to matrix stiffening (Mammoto *et al.* 2022), we proposed that SPP1^high^ macrophages may be sensitive to matrix stiffness.

SPP1 was originally identified as a matrix protein that binds hydroxyapatite with high affinity and regulates the adhesion of osteoclasts to the bone matrix (Craig *et al.* 1988; Heinegård *et al.* 1989). The SPP1 protein has been reported as a biomarker for cardiovascular disease including atherosclerosis (Zheng *et al.* 2012), calcification (Grau *et al.* 2012) and hypertension (Stępień *et al.* 2011), all of which are associated with ECM remodeling of the vessel wall. However, the mechanisms regulating SPP1 expression and secretion remain unknown. In cancer research, SPP1 has been shown to respond to matrix stiffness, with higher levels observed in stiffened tumor regions compared to adjacent tissues (Ghasemi *et al.* 2021). You *et al*. demonstrated that increased matrix stiffness induced higher SPP1 expression via integrin signaling in liver cancer cells (You *et al.* 2015), indicating a mechanical response of SPP1. Here we constructed an *in vitro* PA gel model that mimicks varying stiffness of the vessel wall, revealing that SPP1 expression and secretion were significantly increased in macrophages cultured on stiffer gel. To the best of our knowledge, this is the first study reporting the response of SPP1 expression to matrix stiffness in macrophages.

Imaging flow cytometry (IFC) is an immunological technique by which many cellular parameters can be measured in a robustly quantitative manner (Vis *et al.* 2020). Unlike conventional flow cytometry, IFC enables the acquisition of microscopic images that indicate the position and intensity of proteins in each cell (Hewitt *et al.* 2017). Thus, IFC is a powerful technique for high-throughput quantification of immunofluorescence imaging. In this study, we developed an IFC panel to investigate the mechanical response of SPP1 and found that both the protein intensity, and secretion of SPP1 were elevated in macrophages. Our work presents a novel method that integrates imaging and quantitative analysis to study protein dynamics at the single-cell level.

In conclusion, we integrated bioinformatic analysis with imaging flow cytometry to identify novel cell populations sensitive to matrix stiffness. We discovered that SPP1^high^ macrophages represent a highly active population that regulates ECM composition in stiffened human carotid plaques; Additionally, the expression and secretion of SPP1 in macrophages were modulated by matrix stiffening (Fig. 7E). Our findings provide insight into the mechanism underlying increased SPP1 production during arterial stiffening, and offer a strategy for screening mechano-sensitive cell population.

## METHODS

### Single-cell RNA-seq analyze

The raw data of single-cell RNA sequencing (scRNA-seq) were obtained from the GEO dataset which contains carotid plaques from four patients who underwent endarterectomy. The data was analyzed in RStudio via the standard Seurat workflow. Briefly, the raw data was filtered to exclude low-quality cells with <200 expressed genes and low-expressed genes that are expressed in less than one cell, the mitochondrial gene expression was less than 20%; Subsequently, the DoubletFinder package was used to identify potential doublets (McGinnis *et al.* 2019). To obtain the normalized gene expression data, library size normalization was processed using the NormalizeData function. The top 2000 highly variable genes (HVGs) were calculated using the Seurat function FindVariableGenes. To remove the batch effects in single-cell RNA-sequencing data, the mutual nearest neighbors (MNN) was performed with the R package Batchelor (version 1.6.3) according to previously reported (Haghverdi *et al.* 2018). Graph-based clustering was performed to cluster cells according to their gene expression profile with the FindClusters function. Cells were visualized using RunUMAP and RunTSNE functions. The developmental pseudotime was determined with the Monocle2 (Trapnell *et al.* 2014) package, and cell communication analysis was performed using the CellChat R package (Jin *et al.* 2021).

### Cell culture

RAW 264.7 cells (the murine-derived macrophage cell line) were cultured in high-glucose Dulbecco’s modified Eagle’s medium (DMEM) containing 10% fetal bovine serum. Mouse bone marrow cells were obtained from 10-week-old C57BL/6J mice by flushing the femurs and tibias and were seeded on culture dishes in L929-conditioned medium for seven days to differentiate into bone marrow derived macrophages (BMDM). Cells were cultured in a humidified incubator with 5% CO_2_ at 37°C.

### Animal model

All animal studies were performed in accordance with the guidelines of the Animal Care and Use Committee of Peking University and approved by the Ethics Committee of Peking University Health Science Center (DLASBD0657). In a model of acute aortic calcification, 12-week-old C57/BL6 mice were anesthetized and subjected to a laparotomy for exposure to infrarenal abdominal aortas. Sterile cotton gauze (∼1 cm in length) soaked in 0.5 mol/L CaCl_2_ or saline was applied to the abdominal aortas and then incubated with the aortas for 8 min to induce acute aortic injury. Seven days after the operation, mice were sacrificed and the CaCl_2_ or saline incubated abdominal aortas were harvested for analyses. Considering that men have an increased risk of developing cardiovascular calcification compared to women (Simard *et al.* 2017), male mice were used for this study as previously described (Wang *et al*. 2022).

### PA gel preparation

The PA gels were prepared by mixing 40% *w*/*v* acrylamide and 2% *w*/*v* bis-acrylamide stock solutions and supplemented with N, N, N9, N9-tetramethylethylenediamine (TEMED) and ammonium persulfate to initiate gel polymerization. Gels with different stiffness were obtained by varying the final concentrations of acrylamide (4% and 8%) and bis-acrylamide (0.3% and 0.264%) for the corresponding elastic moduli of ∼2.5 and ∼18.8 kPa, which were selected to mimic the stiffness of healthy and atherosclerotic lesions in human carotid arteries according to published literature (Klein *et al.* 2009; Tse and Engler 2010; Wang *et al.* 2022). 0.5 mg/mL sulfo-SANPAH was incubated on the surface of the gel to cross-link matrix proteins. 0.1 mg/mL collagen type I was coated on the activated gel surfaces. Cells were plated on collagen-coated PA gels for 24 h and then collected for further analysis.

### Measurement of material stiffness

The elastic moduli of PA gel stiffness were measured by nanoindentation. The ferrule-top nanoindenter setup together with the PIUMA controller/drive (Optics11, Amsterdam; The Netherlands) was used and material stiffness was measured as previously described (Mattei *et al.* 2015; Xie *et al.* 2018). During indentation, a probe with a 0.049 N/m spring constant and a spherical tip with a radius of 9 μm was used, the indents were depth controlled (10 μm), and the loading and unloading period was set to be 2 s. 20 to 30 measurements were made on the surface of each material and the Young's modulus were recorded.

### Imaging Flow Cytometry

Flow imaging analysis was performed according to the previously described (Hewitt *et al.* 2017). Briefly, the experiments were performed using a Luminex Mark II platform equipped with 405, 488 and 642 nm lasers for excitation. IDEAS software (Amnis Seattle, WA, USA) was used for analysis. Cells were stained with CD68, SPP1 primary antibody, followed by staining with DAPI and the second fluorophore-conjugated antibody. An unstained tube was prepared alongside test samples as a negative control. Cells were filtered through a 70 μm nylon cell strainer prior to acquisition. A minimum of 20,000 events per sample were acquired using the 40X objective for protein intensity analysis and 60× for protein co-localization analysis. The unstained negative control tube was used to set the laser power, all channels were collected with the laser power set to 16.00 mW for the 488 nm laser, 6.00 mW for the 405 nm scatter laser, and 70.00 mW for the 642 nm laser. Data analysis is detailed in the results section. Briefly, cells were first plotted in the brightfield images to obtain a focused single-cell population, followed by CD68, Rab6A and SPP1 gates based on the intensity of the respective fluorophore channels.

### Immunofluorescence

For cell immunofluorescence assay, Macrophages grown on PA gels were fixed with 4% paraformaldehyde for 30 min. After being blocked with 3% BSA, cells were incubated with primary antibody against SPP1 over night at 4°C. After incubation with the second antibody and Phalloidin, cells were mounted with a mounting medium containing DAPI and visualized by confocal microscopy equipped with Airyscan mode (ZEISS LSM 900) to obtain super-resolution images (Huff 2015). The immunofluorescence of mice vessel sections was similar to that in cultured cells. Quantification of staining was conducted by using NIH ImageJ software.

## Conflict of interest

Jin Wang, Jiyin Wang, Yuting Zhang, Chaoyang Xiong, Shun-Ai Liu, Yaxian Kong, Jing Zhou and Xi Wang declare that they have no conflict of interest.
